# P4-ATPase subunit Cdc50 plays a role in yeast budding and cell wall integrity in *Candida glabrata*

**DOI:** 10.1186/s12866-023-02810-3

**Published:** 2023-04-13

**Authors:** Ke-Zhi Chen, Lu-Ling Wang, Jin-Yan Liu, Jun-Tao Zhao, Si-Jia Huang, Ming-Jie Xiang

**Affiliations:** 1 Department of Laboratory Medicine, Ruijin Hospital, Shanghai Jiao Tong University School of Medicine, Shanghai, China; 2 Department of Laboratory Medicine, Ruijin Hospital Luwan Branch, Shanghai Jiao Tong University School of Medicine, Shanghai, China

**Keywords:** *Candida glabrata*, Lipid flippase, Cdc50, Drug susceptibility, Cell wall integrity, Virulence

## Abstract

**Background:**

As highly-conserved types of lipid flippases among fungi, P4-ATPases play a significant role in various cellular processes. Cdc50 acts as the regulatory subunit of flippases, forming heterodimers with Drs2 to translocate aminophospholipids. Cdc50 homologs have been reported to be implicated in protein trafficking, drug susceptibility, and virulence in *Saccharomyces cerevisiae*, *Candida albicans* and *Cryptococcus neoformans*. It is likely that Cdc50 has an extensive influence on fungal cellular processes. The present study aimed to determine the function of Cdc50 in *Candida glabrata* by constructing a *Δcdc50* null mutant and its complemented strain.

**Results:**

In *Candida glabrata*, the loss of Cdc50 led to difficulty in yeast budding, probably caused by actin depolarization. The *Δcdc50* mutant also showed hypersensitivity to azoles, caspofungin, and cell wall stressors. Further experiments indicated hyperactivation of the cell wall integrity pathway in the *Δcdc50* mutant, which elevated the major cell wall contents. An increase in exposure of β-(1,3)-glucan and chitin on the cell surface was also observed through flow cytometry. Interestingly, we observed a decrease in the phagocytosis rate when the *Δcdc50* mutant was co-incubated with THP-1 macrophages. The Δ*cdc50* mutant also exhibited weakened virulence in nematode survival tests.

**Conclusion:**

The results suggested that the lipid flippase subunit Cdc50 is implicated in yeast budding and cell wall integrity in *C. glabrata*, and thus have a broad influence on drug susceptibility and virulence. This work highlights the importance of lipid flippase, and offers potential targets for new drug research.

## Background

As the most common opportunistic fungi, *Candida* species are usually part of the human normal flora, yet retain the ability to cause both mucosal infection (e.g., thrush and vulvovaginal candidiasis) and invasive candidiasis under certain conditions, such as improper usage of antifungal drugs or immunodeficiency virus infection [1–4]. In recent years, a worldwide increase in the prevalence of invasive candidiasis has been observed, which was mostly attributed to expanded immunocompromised populations [5–7]. Among all *Candida* species, *Candida glabrata* accounts for nearly 21% of the *Candida* bloodstream infections in Canada [8] and 24% of the systemic infections among USA transplant recipients [5], ranking only second to *Candida albicans*. Possessing the highest mortality rate among *Candida* species, hematogenous candidiasis caused by *C. glabrata* has remained a severe problem [9, 10]. Additionally, *C. glabrata* has been reported to quickly develop adaptation to moderate fungicidal azoles exposure [11–13]. However, there’s no sufficient understanding concerning what makes *C. glabrata* the second most infectious *Candida*, considering that it lacks the yeast-hyphal switch or secreted aspartyl proteases like other *Candida* species [1, 2, 13]. More attention should be paid to *C. glabrata* to determine its pathogenic mechanism, with the aim of minimizing the damage it might cause.

P4-ATPases, a class of lipid flippases that drive the ATP-dependent translocation of aminophospholipids in eukaryotic cells, have attracted great attention in recent years [14–17]. P4-ATPases play a crucial role not only in physiological processes, but also in some clinically significant diseases. For example, ATP10A, a mutant form of human P4-ATPase, is implicated in type 2 diabetes, and ATP11A is implicated in tumor progression [14]. The broad influence of P4-ATPases is at least partly caused by their function in maintaining the asymmetric lipid distributions between eukaryotic membrane bilayers [18, 19]. Notably, the compositions of each leaflet are not symmetrically distributed: Phosphatidylcholine (PC) and sphingolipids are mostly located in the exoplasmic or luminal leaflet, while amino phospholipids (phosphoserine (PS) and phosphatidylethanolamine (PE)) occupy the cytoplasmic leaflet [18, 20, 21]. Such lipid gradients help in membrane budding and fission, thereby mediating various cellular processes, including secretory protein trafficking, cargo sorting, and signal transduction [21, 22].

Fungal P4-ATPases have been characterized in several previous studies. Consisting of α catalytic and β regulatory subunits, P4-ATPases are highly conserved among *Saccharomyces cerevisiae* and *Candida* species [21, 23]. To date, three homologs have been found in baker’s yeast and *Candida* species: the Drs2-Cdc50 complex, the Dnf1/Dnf2-Lem3 complex, and Neo1 [24, 25]. Saito et al. [26, 27] first characterized the fungal Drs2-Cdc50 complex in *S. cerevisiae*, demonstrating that Drs2 and Cdc50 depended on each other to transport from the endoplasmic reticulum (ER) to the trans-Golgi network (TGN) membrane. The yeast *Δcdc50* mutant showed defects in protein transport, actin organization, and vacuole structure in cold temperatures [27]. A screening test in 2016 [28] also found the loss of Cdc50 led to increased sensitivity to caspofungin in *Cryptococcus neoformans*. Furthermore, Cdc50 was reported to mediate azole susceptibility, cell wall and membrane integrity, and the response to pH and hyperosmotic stresses in *Cryptococcus neoformans* [28–30]. In the dimorphic fungi *Candida albicans*, besides having a hypersensitive phenotype to terbinafine, caspofungin, and azoles, the *Δcdc50* mutant also possessed defective hyphal growth and virulence toward mice [23]. Given its strong conservation among yeast-like fungi, the Cdc50 homolog presumably plays a similar role in *C. glabrata*.

In the present study, to determine the function of Cdc50, we constructed a *Δcdc50* null mutant of *Candida glabrata* standard strain ATCC2001 via homologous recombination and a *Δcdc50* + *CDC50* complemented strain based on the null strain. The results implied that Cdc50 is required for yeast budding and cell wall integrity in *C. glabrata*, and thus have a broad influence on drug resistance and virulence Furthermore, the cell wall integrity (CWI) pathway and downstream effector genes associated with cell wall biogenesis and remodeling were found to be constitutively upregulated in the *Δcdc50* mutant.

## Results

### Loss of Cdc50 causes a moderate defect in cell growth and yeast budding in *C. glabrata*

**Fig. 1:**
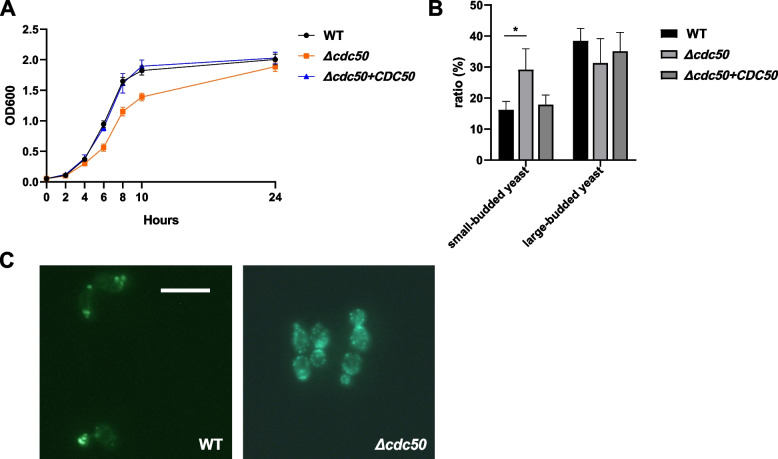
The *Δcdc50* mutant showed defects in cell growth, yeast budding, and actin polarization. **A** Wild-type (WT), *Δcdc50*, and *Δcdc50* + *CDC50* strains were adjusted to the same cell density and incubated in fresh YPD medium at 30 ℃ for 24 h. Their OD at 600 nm was recorded to construct the cell growth curve. In the exponential phase, the *Δcdc50* mutant exhibited significantly slower growth compared with the other two strains. **B** The cell cycle distribution of different strains as determined using microscopy. Mid-log phase yeast was collected and stained to be classified as having no, small, or large buds. More than 300 cells were counted in each group. The *Δcdc50* mutant showed increased numbers of small buds and decreased numbers of large buds compared with those of the wild-type. Data represent the mean ± SD of three independent experiments. **C** Typical views of wild-type and *Δcdc50* actin fluorescent imaging during yeast budding. After staining with FITC**-**phalloidin, the WT and *Δcdc50* yeast cells were observed using fluorescence microscopy. Most WT budding yeasts exhibited normal actin patch polarization (enriched in buds and bud necks), while a large amount of *Δcdc50* budding yeasts showed actin mislocalization: The actin patches were depolarized and scattered. Bar, 10 μm. *, *P* < 0.05. Data represent the mean ± SD of three independent experiments

Given that P4-ATPase subunit Cdc50 homologs in other common pathogenic fungi have a significant influence on cellular processes, a *Δcdc50* null mutant and its complemented strain were constructed to study how it functions in *Candida glabrata*. First, the wild-type (WT), *Δcdc50*, and complemented strain *Δcdc50* + *CDC50* were incubated for 24 h, and their optical density (OD) at 600 nm was recorded to construct a growth curve (Fig. 1A). As the growth curve indicated, the *Δcdc50* mutant showed defective cell growth, especially in exponential phase, during which other strains grew rapidly. However, after 24 h of incubation, the WT and *Δcdc50* mutant ended up with similar optical densities, despite the OD of the WT being almost as twice that of *Δcdc50* at 6 h. This implies that proper functioning of Cdc50 is required for rapid cell growth in *C. glabrata*.

Next, to demonstrate what causes the defective growth in the *Δcdc50* mutant, we examined the cell cycle distributions of the WT and *Δcdc50* mutant by microscopic observance, as shown in Table 1 and Fig. 1B. In the *Δcdc50* mutant, the proportion of small-budded yeast increased from nearly 16% to 29% (*P* < 0.05), while large-budded yeast showed a slight decrease from 38 to 31%. The accumulation of small buds in cell cycle suggests that some of the *Δcdc50* mutant cells have a difficulty in bud growth, which might be responsible for the observed cell growth defect.

**Table 1:**
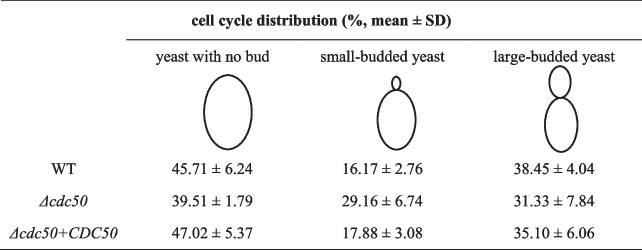
Cell cycle distributions

As an essential cellular process for reproduction, yeast budding relies on the normal function of various accessory proteins, among which actin polarization plays a great part [31, 32]. During mitosis, actin undergoes rapid assembly and polarization to provide forces for morphogenesis. Disorganization of the actin cytoskeleton might easily disrupt bud growth and lead to cell cycle arrest. Actually, the budding block has been characterized in the *S. cerevisiae Δcdc50* mutant by Saito et al. [27], in which they attributed the yeast growth defect to actin patch depolarization and actin cable disappearance caused by mislocalization of proteins associated with actin organization, e.g., Bni1. Thus, to determine whether the actin depolarization also occurred in the *C. glabrata Δcdc50* mutant, actin staining using fluorescein isothiocyanate (FITC)-phalloidin was performed (Fig. 1C). Fluorescent imaging showed that most of the WT budding yeast exhibited normal actin patch polarization (enriched in buds and bud necks), while a large amount of *Δcdc50* budding yeast showed actin mislocalization, in which the actin patches were depolarized and scattered. These morphological observations suggested that the *Δcdc50* mutant exhibited a growth defect and budding block, which might be at least partly caused by actin depolarization. For further validation, subcellular localization and null strain construction of the essential factors of cell polarity, such as Cdc42, may be needed.

### The *Δcdc50* mutant exhibits hypersensitivity to azloes and caspofungin

**Fig. 2:**
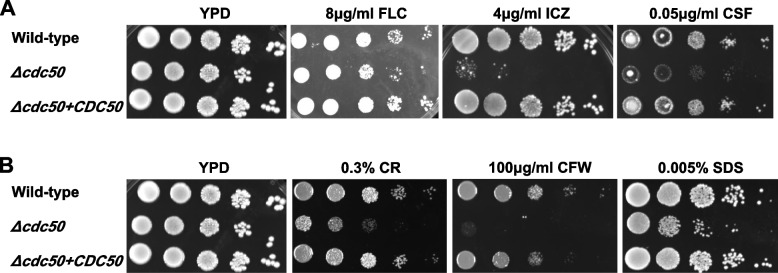
The *Δcdc50* mutant showed hypersensitivity to antifungal drugs and cell wall stressors. Mid-log phase yeast was adjusted to 0.1 OD at 600 nm and diluted tenfold serially to be spotted on YPD plates containing different drugs. **A** The *Δcdc50* mutant exhibited weakened resistance to the clinically used antifungal drugs fluconazole, itraconazole and caspofungin. FLC, fluconazole; ICZ, itraconazole; CSF, caspofungin. **B** The *Δcdc50* mutant was more sensitive to cell wall stressors CR, CFW and SDS, suggesting cell wall remodeling. CR, Congo Red; CFW, Calcofluor White; SDS, sodium dodecyl sulfate

Azoles, polyenes and echinocandins are the most commonly used antifungal drugs. Considering that losing Cdc50 in other common yeasts has been reported to cause hypersensitivity to multiple drugs, both spot assays and reference methods of broth dilution were used to assess whether *C. glabrata* is required for surviving from azoles, amphotericin B, and caspofungin. As presented in Fig. 2A, the *Δcdc50* mutant was hypersensitive to itraconazole and caspofungin, but displayed only slightly changed sensitivity to fluconazole, compared with that in the WT strain, and re-integrating *Cdc50* restored drug resistance.

Furthermore, we performed broth microdilution tests in Roswell Park Memorial Institute (RPMI)1640 liquid medium, the recommended method of the Clinical and Laboratory Standards Institute (CLSI), to quantify the drug susceptibility of different strains (Table 2). We found that the 50% minimum inhibitory concentration (MIC50) of fluconazole, itraconazole, voriconazole and caspofungin in the WT was respectively as four, four, two and eight times that in the *Δcdc50* mutant, which agreed with the results of the spot assays. This evidence indicated that the cellular defects of the *Δcdc50* mutant might affect the establishment of drug tolerance. Interestingly, for amphotericin B, there was no significant difference between the susceptibility of the WT and *Δcdc50* strains, whether on plates (data not shown) or in RPMI1640 broth (Table 2).

**Table 2:** MIC50 of different strains in RPMI1640 broth

	MIC50 of strains at 48 h in RPMI1640 broth (μg/ml)		
Drug	WT	*Δcdc50*	*Δcdc50* + *CDC50*
fluconazole	1	0.25	1
itraconazole	0.125	0.031	0.125
voriconazole	0.25	0.125	0.25
caspofungin	0.125	0.016	0.125
amphotericin B	0.5	0.5	0.5

In terms of antifungal mechanisms, azoles target and suppress the ergosterol biogenesis pathway, an important process for the fungal plasma membrane, to cause membrane structure damage [11], while echinocandins specifically inhibit fungal β-(1,3)-glucan synthases to induce deficient cell walls lacking β-(1,3)-glucan, which fails to shelter cells from multiple outside stresses [34]. Increased sensitivity to azoles and caspofungin observed in the *Δcdc50* mutant suggested some structural defects in cell membrane and cell wall, respectively.

### Cdc50 disruption led to cell wall remodeling in *C. glabrata*

Since the *Δcdc50* mutant possessed hypersensitivity to azoles and caspofungin compared with the WT strain, YPD plates containing 0.005% sodium dodecyl sulfate (SDS), 0.3% Congo Red and 100 μg/ml Calcofluor White (CFW), respectively, were used for stress assays. Among these stressors, SDS works as a surfactant to dissolve the yeast cell membrane, which shows enhanced fungistatic activity with the existence of cell membrane or wall damage. Congo Red and CFW are chitin-binding agents often used to estimate cell wall integrity [35]. As shown in Fig. 2B, after growing for 48 h, the *Δcdc50* mutant exhibited severe hypersensitivity to Congo Red and CFW compared with the WT and complemented strain, implying an altered cell wall in the *Δcdc50* mutant. Besides, *Δcdc50* yeast seemed to be weaker in response to SDS exposure, probably due to increased permeability of the cell wall.

As a highly-conserved structure in fungi, the cell wall plays a vital role, acting as a structural support and primary shield from various external stresses, such as mechanical, osmotic, oxidative and pH stresses [36]. It is composed of the inner β-(1,3)-glucan and chitin layer, which are the two main pathogen-associated molecular patterns (PAMPs) for host innate immune cell recognition, as well as the outer mannan skeleton and highly mannosylated protein branches that mask the inner layer, avoid recognition, and minimize host immune responses [2, 35, 37]. Any shortage in these compositions or change in structure may result in weakened stress resistance.

### Loss of Cdc50 leads to increased cell wall components

**Fig. 3:**
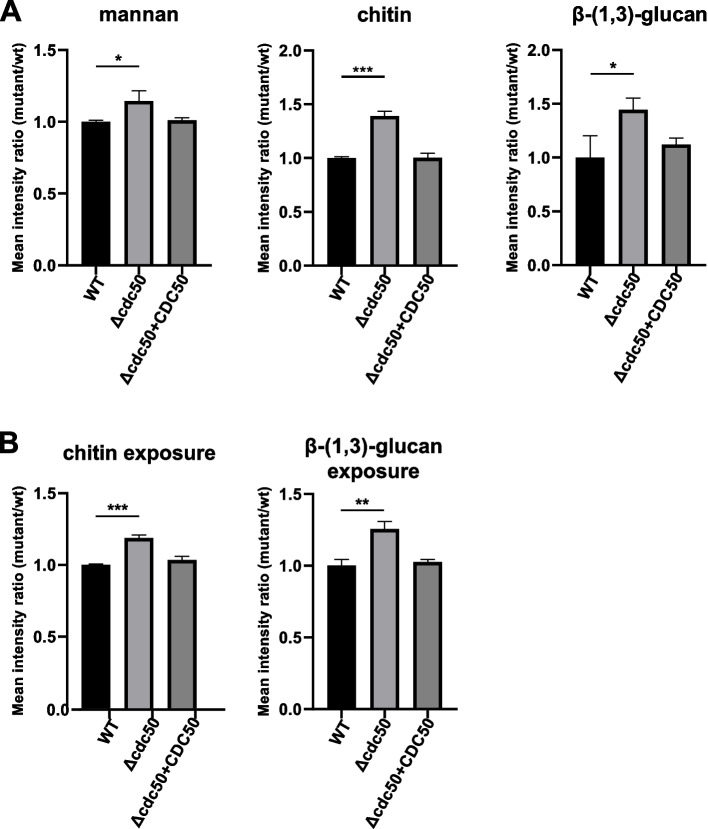
Increased cell wall mannan, chitin and β-1,3-glucan contents, along with exposed chitin and β-1,3-glucan were found in *Δcdc50*. **A** Yeast cells in mid-log phase were collected and incubated in FITC-ConA, CFW and Aniline Blue to assess the total cell wall mannan, chitin, and β-1,3-glucan contents, respectively, then analyzed using flow cytometry and spectrofluorometry. In the *Δcdc50* mutant, the cell wall mannan content increased by 14.5%, total chitin contents increased significantly by 39%, and total β-1,3-glucan contents increased by 44.3%. **B** Exposure of chitin and β-1,3-glucan were tested using FITC-WGA and anti-β-1,3-glucan antibodies. The exposure of inner chitin has increased by 14.9%, while the β-1,3-glucan exposure has increased by 25.5% in the *Δcdc50* mutant. Data represent the mean ± SD of three independent experiments. *, *P* < 0.05; **, *P* < 0.01; ***, *P* < 0.001

To determine the reason for the defective cell wall in the *Δcdc50* mutant, different fluorescent dyes combined with flow cytometry and spectrofluorometry were used to assess the cell wall compositions. FITC conjugated lectin from Concanavalin A (ConA), CFW and Aniline Blue were used for total mannan, chitin and β-(1,3)-glucan measurements, respectively. The data acquired was analyzed and the mean fluorescence intensities are presented in Fig. 3A. Interestingly, according to the results, all the major compositions of the *Δcdc50* mutant were higher than those in the WT: The mannan content increased by 14.5% (*P* < 0.05), the chitin content increased by 39% (*P* < 0.001), and the β-(1,3)-glucan content increased by 44.3% (*P* < 0.05), indicating that losing Cdc50 might lead to a thicker cell wall in *C. glabrata*. Additionally, the increased cell wall chitin contents might be responsible for the CFW-sensitive phenotype of *Δcdc50*, as in the majority of cases, increased chitin was thought to be connected with hypersensitivity to CFW [38].

### An increase in β-(1,3)-glucan and chitin exposure was detected on the *Δcdc50* mutant cell surface

We have found that the total mannan, chitin and β-(1,3)-glucan contents in the *Δcdc50* mutant were higher than those in the WT and complemented strain. However, since the inner cell wall layer is mostly masked behind the outer mannan layer, it is the exposure of glucan and chitin, not the total content that has the most influence on the host-*Candida* interaction [2, 37], which highlights the importance of exposure detection. FITC-conjugated wheat germ agglutinin (WGA), which binds N-acetylglucosamine specifically, was used to estimate the chitin exposure, while an anti-β-(1,3)-glucan monoclonal antibody was used to measure exposed β-(1,3)-glucan, as the dye and antibody molecules are too large to pass through the mannan skeleton and are only capable of binding to the exposed part of inner layer [39, 40]. The data acquired via flow cytometry showed that the exposure of β-(1,3)-glucan in the *Δcdc50* mutant was increased by 25.5% (*P* < 0.01), and the exposure of chitin increased by 18.7% (*P* < 0.001) (Fig. 3B), which demonstrated that disrupting Cdc50 promoted unmasking of β-(1,3)-glucan and chitin on the cell surface.

### The *Δcdc50* mutant shows less uptake and enhanced pro-inflammatory cytokine secretion by macrophages

**Fig. 4:**
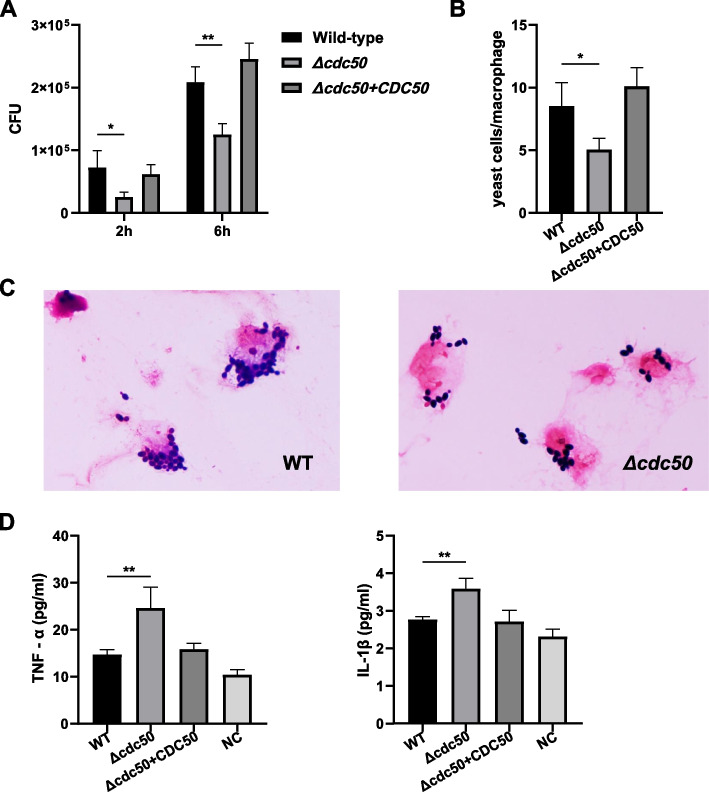
Loss of Cdc50 led to decreased phagocytosis rate in macrophages and increased pro-inflammatory cytokine secretion. **A** Aliquots of THP-1 cells were co-incubated with wild-type (WT), *Δcdc50*, and *Δcdc50* + *CDC50* yeast for 2 h, then washed and lysed to release intracellular yeast for CFU counting. Other THP-1 cells were cultured for another 4 h after the 2-h co-incubation and washing, and lysed for CFU counting. After being exposed to macrophages for 2 h, the *Δcdc50* mutant exhibited a much lower phagocytosis rate compared with the WT and *Δcdc50* + *CDC50* strains. Accordingly, the number of surviving intracellular *Δcdc50* yeast were reduced by almost 50% compared with that of the surviving WT yeasts after 6 h. **B** After a 2-h co-incubation with yeast cells, THP-1 cells were brushed and Gram stained to count the intracellular yeast. The *Δcdc50* cells were about 58.6% of WT cells, exhibiting less uptake by macrophages. **C** Typical field views of THP-1 after co-incubation with WT and *Δcdc50* cells, respectively. **D** Different *C. glabrata* strains were co-incubated with THP-1 cells for 4 h, and the culture supernatants were collected and centrifugated to measure the secreted TNF-α and IL-1β levels. THP-1 cells without exposure to yeast were used for baseline secretion level estimation as a negative control. Exposure to WT and *Δcdc50* + *CDC50* hardly triggered enhanced secretion of pro-inflammatory cytokines in macrophages, whereas the loss of Cdc50 activity in *C. glabrata* caused increased secretion of TNF-α and IL-1β. Data represent the mean ± SD of three independent experiments. *NC* Negative control. *, *P* < 0.05; **, *P* < 0.01

Increased β-(1,3)-glucan and chitin exposure on the cell surface is considered as a signal for innate immune cell recognition and phagocytosis, especially by macrophages. The unmasking of β-(1,3)-glucan and chitin in the *Δcdc50* mutant suggested that Cdc50 might affect the interaction with host immune cells through cell wall alternations. Therefore, phagocytosis assays for THP-1 macrophages co-incubated with *C. glabrata* were performed, using both yeast counting and pro-inflammatory cytokine measurements to determine the yeast's ability to escape from phagocytosis and their survival and proliferation inside macrophages. First, plate colony forming units (CFU) counting after a 2-h co-incubation was performed. Surprisingly, the *Δcdc50* mutant was subjected to much less phagocytosis (35%) than the WT (Fig. 4A) (*P* < 0.05). Moreover, after washing away the extracellular yeast, another plate was cultured for a further 4 h to detect the yeast proliferation capacity. The number of *Δcdc50* mutant cells was only 60% compared with the WT cells (*P* < 0.01; Fig. 4A).

To further validate the decreased phagocytosis rate of the *Δcdc50* mutant and eliminate the effect of Triton usage, we conducted the microscopic yeast counting. Macrophages after co-incubating with yeast for 2 h were brushed from the plate and Gram stained to count the intracellular yeast cells. As shown in Fig. 4B, the intracellular WT and complemented cells inside the macrophages were a little more than *Δcdc50* cells (P < 0.05), and the typical views of WT and *Δcdc50* were presented in Fig. 4C. As *Candida glabrata* is known for its survival and proliferation in macrophages to avoid other immune cells, especially neutrophil-mediated killing, the decrease in uptake by THP-1 cells may result in less surviving *C. glabrata* in the host [41].

In addition to direct phagocytosis, macrophages also secrete pro-inflammatory cytokines to recruit more immune cells in the immune response against *Candida* species. However, *C. glabrata*, among all the *Candida* species, is known for minimizing cytokine secretion and suppressing inflammatory responses for immune escape [42, 43]. Therefore, we tested the levels of tumor necrosis factor alpha (TNF-α) and interleukin 1 beta (IL-1β) cytokines in the THP-1 cell supernatant using a chemiluminescence method after co-incubating THP-1 and *C. glabrata* for 4 h. The cytokines secreted by THP-1 without exposure to *C. glabrata* were measured as a negative control. We observed an increase in TNF-α and IL-1β secretion by 67.69% (P < 0.01) and by 29.62% (P < 0.01), respectively, in *Δcdc50* mutant (Fig. 4D). Apparently, disruption of Cdc50 weakened the ability of *C. glabrata* to suppress THP-1 macrophages from secreting pro-inflammatory cytokines.

According to the results of CFU counting and cytokine measurements, we concluded that the *Δcdc50* mutant showed attenuated immune escape, with decreased macrophage uptake and increased secretion of pro-inflammatory cytokines.

### The* Δcdc50* mutant shows weakened virulence toward *Caenorhabditis elegans*

**Fig. 5:**
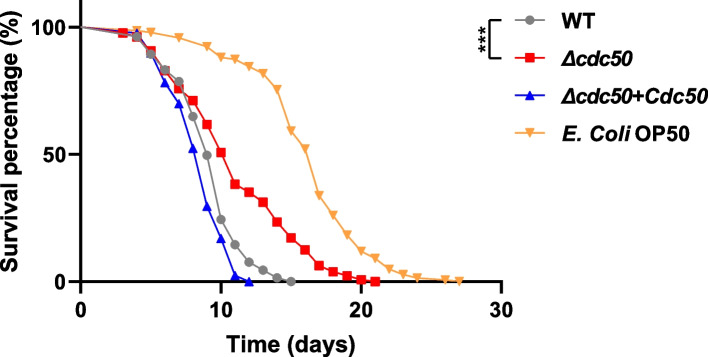
The *Δcdc50* mutant showed defective virulence in *Caenorhabditis elegans* compared with other strains. *C. elegans* were raised and fed on different strains of *C. glabrata*, and the number of deaths in each group was counted daily to construct a survival curve and assess in vivo virulence. *E. coli* OP50 strain was used as a quality control. Nematodes in the *Δcdc50* group exhibited a longer lifespan compared with the wild-type (WT) and *Δcdc50* + *CDC50* group, suggesting weakened virulence in the *Δcdc50* mutant. ***, *P* < 0.001

We observed that Cdc50 is associated with cell wall integrity, which is of vital importance of fungal virulence; therefore, we carried out *C. elegans* survival assays to estimate the overall virulence of the *Δcdc50* mutant in vivo. As a classical model organism that feeds on microorganisms, *C. elegans* is often used to evaluate the virulence of fungal and bacterial pathogens [44]. Over 300 *C. elegans* were transferred to plates containing medium supporting *C. glabrata* growth, which were incubated for at least 25 days, during which the death count of each group was recorded daily to construct the survival curve shown in Fig. 5, and *Escherichia coli* OP50 strain was used as a quality control. In general, the lifespan of *C. elegans* feeding on *Δcdc50* mutant was significantly longer compared with that for nematodes feeding on WT and *Δcdc50* + *CDC50* yeast (*P* < 0.001). Although *C. elegans* in different groups shared similar medium lifespans, thereafter nematodes exposed to the *Δcdc50* yeast cells exhibited a much longer maximal lifespan. Disrupting Cdc50 allowed a larger number of nematodes to endure for a longer time, which might have something to do with the defective proliferation capacity that grows more and more visible over time, due to budding block and cell wall defect described before.

Overall, we conclude that losing Cdc50 led to weakened virulence of *C. glabrata* toward *C. elegans*.

### The cell wall integrity (CWI) pathway is constitutively activated in the *Δcdc50* mutant

*Candida* species have evolved several highly-conserved mechanisms in response to various external stresses, one of which is the CWI pathway. The whole CWI pathway consists of cell surface sensors, GTP/GDP exchange factors (GEFs), Rho family GTPases, protein kinases C (PKCs), the mitogen-activated protein kinase (MAPK) cascade, downstream transcription factors, and effectors [37]. To investigate the mechanism of cell wall remodeling in the *Δcdc50* mutant, both quantitative real-time reverse transcription PCR (qRT-PCR) and transcriptome sequencing (RNA-sequencing (RNA-seq)) were used to estimate the mRNA expression levels of MAPKs, downstream transcription factors and effectors associated with cell wall biogenesis.

**Fig. 6:**
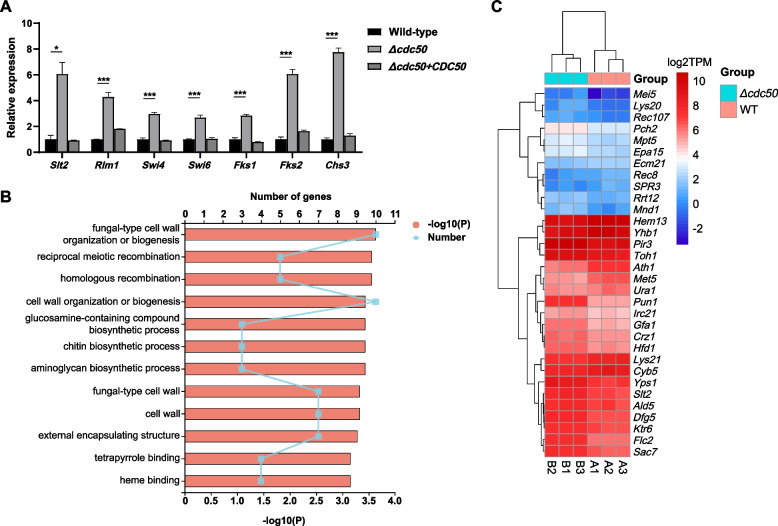
Stress response pathways associated with cell wall biogenesis and remodeling were constitutively upregulated in the *Δcdc50* mutant. **A** Mid-log phase yeast was collected, washed, and frozen to isolate total RNA, followed by qRT-PCR. The relative expression of genes in different strains were obtained by comparison with that in the wild-type (WT). In the *Δcdc50* mutant, the genes that encode the constituents of the cell integrity pathway, including *Slt2*, *Rlm1*, *Swi4*, *Swi6*, *Fks1*, *Fks2* and *Chs3*, all showed increased expression to varying degrees. **B** Mid-log phase WT and *Δcdc50* yeast were collected for complex transcriptome sequencing, and the expression level of every gene was quantified using the TPM method. Differentially expressed genes were enriched via GO functional enrichment analysis to identify significantly different GO pathways between the WT and *Δcdc50*. The top 12 most significant pathways were mainly implicated in cell wall organization and biogenesis. **C** The TPM of genes enriched in the GO pathways in **B** were gathered and taken the logarithm to generate a heatmap for visualized comparison. TPM, transcripts per million reads; GO, Gene Ontology. *, *P* < 0.05; **, *P* < 0.01; ***, *P* < 0.001

Through qRT-PCR, we found that *Slt2,* encoding the already characterized MAPK in *C. glabrata* [45, 46], is constitutively upregulated in the *Δcdc50* mutant, with an almost six-fold increase in transcription (Fig. 6A) (*P* < 0.05). The increasing trend was also seen in its downstream transcription factor-encoding genes *Rlm1*, *Swi4* and *Swi6*, whose expression levels increased by 429%, 298%, and 269% respectively (all *P* < 0.001). The cell wall biogenesis genes were also transcriptionally hyperactivated, with β-(1,3)-glucan synthase-encoding *Fks1* increasing by about three-fold, *Fks2* by six-fold, and chitin synthase-encoding *Chs3* by eight-fold (all *P* < 0.001). Thus, it appeared that the CWI pathway is constantly hyperactivated in the *Δcdc50* mutant.

In the RNA-seq data, the differential expression genes (DEGs) between the WT and the *Δcdc50* mutant were analyzed using Gene Ontology (GO) to reveal the pathways implicated in Cdc50 disruption. Unsurprisingly, the top 12 identified pathways were mostly associated with fungal cell wall organization and biogenesis (Fig. 6B). More specifically, 31 genes annotated with top 10 pathways were selected and visually compared in a heatmap (Fig. 6C). According to the heatmap, among these DEGs, *Pir3*, *Toh1*, *Yps1* and *Dfg5*, which all encode glycosyl phosphatidyl inositol (GPI)-anchored proteins, are associated with cell wall integrity, and all of them were transcriptionally activated in the *Δcdc50* mutant, suggesting cell wall-stressed conditions. Moreover, *Sac7*, whose homolog in *S. cerevisiae* encodes a Rho family GTPase activating protein, and *Slt2*, both showed upregulated expression, which demonstrated activation of the CWI pathway. Interestingly, *Crz1*, encoding the downstream transcription factor of the calcineurin pathway, and *Flc2*, encoding the putative calcium channel protein involved in stress responses, were also upregulated, which might be considered as a sign of global stress response induction in *Δcdc50* mutant.

Altogether, the transcriptional evidence from qRT-PCR and RNA-seq correspond with the increase in cell wall components mentioned above, indicating that cell wall biogenesis and remodeling of the *Δcdc50* mutant is constitutively hyperactivated, and stress response pathways are upregulated.

## Discussion

Among all the common *Candida* species, *Candida glabrata* is rather distinctive. It possesses no secreted hydrolases or pseudohyphae structures for invasive growth, let alone the capacity for white-opaque switching [1, 13]. Yet it remains an evolutionarily successful opportunistic pathogen, contributing to 10–20% of invasive candidiasis in Europe [4], with a 50% mortality rate among bloodstream infection populations, suggesting we should pay more attention to the underlying virulence factors. Meanwhile, acquired drug resistance in *Candida* species during antifungal treatment has risen to be a severe problem worldwide in recent decades [11, 12]; narrow range of antifungal drug classes has made this situation worse. Thus, further investigations into virulence factors or potential new antifungal targets are warranted.

Cdc50, a regulatory subunit of the P4-ATPase lipid flippase, has been reported to be required for protein trafficking, virulence, and azole and echinocandin tolerance in *S. cerevisiae*, *C. albicans* and *C. neoformans* [22, 47]. In *S. cerevisiae*, Cdc50 has been characterized to bind the catalytic subunit Drs2, helping to translocate aminophospholipids between the membrane bilayers to maintain the dynamic lipid asymmetry [26]. The Drs2-Cdc50 complex is localized to the TGN and late endosome membrane, while the homolog P4-ATPase in *S. cerevisiae*, the Dnf1-Lem3 complex, is localized to the plasma membrane, implying a spatial and functional differentiation in lipid flippases [26]. In the present report, we determined that Cdc50 has an influence on yeast budding and drug susceptibility in *C. glabrata*, which corresponds with the results of studies in other fungi, suggesting that P4-ATPases act as evolutionarily conserved proteins in fungi.

As a subunit of lipid flippase, why is fungal Cdc50 so widely influential? Perhaps it acts through the lipid asymmetry that forms the foundation of various cellular processes. On the one hand, the asymmetric distributions of differently charged aminophospholipids provide electronic charge conditions for proteins to bind or dissociate from the leaflet via electrostatic interactions. On the other hand, lipid gradients allow the existence of membrane curvature, enable membrane budding and fusion, and consequently, promote protein transport, secretion and internalization [15]. In the absence of lipid flippases, the membrane system remains stationary, causing trouble for inter- and intra-cellular material communications.

In this study, we first characterized the alterations in actin organization in the *C. glabrata Δcdc50* mutant, which were first recognized in *S. cerevisiae* in 2004 [26]. During the lifecycle of yeast, frequent actin reorganization plays an essential part in morphogenesis, providing support for cellular processes such as vesicle internalization and budding growth. Fast polarization and disassembly of actin cables and patches requires the precise regulation and activation of at least 100 highly conserved accessory proteins [31, 48, 49]. For instance, at bud emergence, Rho family GTPase Cdc42 acts as a key regulator to recruit various effectors, e.g., Bni1 and Gic2, to promote actin cable polarization and actin patch clustering. Therefore, we developed a hypothesis to explain the actin mislocalization that was observed in the *Δcdc50* mutant: By losing lipid flippase activity, the disrupted lipid asymmetry hindered protein trafficking, leading to mislocalization and malfunction of certain accessory protein, thus disturbing actin organization. This was partly supported by the work of Das et al. [50], who reported that losing Lem3, a Cdc50 family protein, led to alterations in the electronic charges of the plasma membrane, thus triggering failure to extract Cdc42 from the membrane.

Hypersensitivity to azoles has been reported in *C. albicans*, *C. neoformans*, and *C. glabrata* after disturbing the function of lipid flippases, exhibiting high conservation among yeasts. Although this susceptibility has received little further research attention, a report published in 2015 [47] offers an explanation. The authors reported that by flipping PS between the membrane bilayers, the Cdc50-Drs2 flippase system in *S. cerevisiae* appeared to play a crucial part not only in cargo protein sorting, but also in ergosterol enrichment in the plasma membrane. Besides, this defect was thought to be achieved by mislocating the oxysterol-binding protein homologue, Kes1/Osh4. Thus, in *C. glabrata*, lipid flippases might also be required to control ergosterol's subcellular distribution by regulating the localization of specific accessory proteins, and the loss of flippase activity might result in a defective membrane, making it vulnerable when exposed to azoles. In agreement with this hypothesis, in *S. cerevisiae*, the synthetical lethality of mutations in *Cdc50* and genes associated with the late steps of ergosterol biogenesis (*Erg2* ~ *6*) were discovered [51]. Besides, hypersensitivity to drugs affecting phospholipid metabolism (cinnamycin and miltefosine) was observed in the *C. neoformans Δcdc50* mutant, implying altered membrane phospholipids [29]. Further studies, including membrane composition analysis combined with accessory protein localization, are required to determine the mechanism underlying this defect.

Another significant phenotype we observed in the *Δcdc50* mutant was cell wall remodeling, which has also been reported in *C. neoformans* [28]. We observed hypersensitivity to Congo Red and CFW, which is usually implicated in increased chitin contents in the cell wall. Further fluorescence staining showed increase in all the three major cell wall components. The increase in the chitin content is thought to correlate positively with tolerance to caspofungin, but the β-(1,3)-glucan content usually correlates negatively with caspofungin tolerance [38, 52]; therefore, the hypersensitivity to caspofungin in the *Δcdc50* mutant seems to be the result of both changes. Moreover, a thicker outer cell wall mannan layer is usually connected with better masking of the inner glucan and chitin; but to our surprise, an increase was observed not only for cell wall mannan contents, but also for the inner chitin and β-(1,3)-glucan exposure on the cell surface. A possible explanation is that loss of Cdc50 causes rearrangements of the cell wall, which changes the mannan structure and results in unmasking of the inner cell wall layer.

For the yeast-macrophage interaction, while most common *Candida* species have evolved a well-masked inner layer for strategic avoidance, *C. glabrata* has evolved a well-regulated remodeling system to survive and reproduce inside macrophages instead [2, 43]. Some researchers even believe that *C. glabrata* favors being taken up by, and proliferating in macrophages to avoid instant killing by neutrophils [41]. Correspondingly, the exposure of inner cell wall glucan and chitin in *C. glabrata* is reported to be naturally much higher than in other *Candida* species [36, 38]. Therefore, the decrease in the phagocytosis rate of the *Δcdc50* mutant was not likely to favor host survival and can be considered as a weakened virulence factor. Nonetheless, it was still surprising that more glucan and chitin exposure on the *Δcdc50* cell surface did not induce increased uptake by macrophages, but this apparent paradox is not unique. In a study published in 2020 [39], in which different clinical *Candida* isolates were treated with sub-MIC concentration caspofungin, strains with increased cell wall glucan and chitin exposure also experienced reduced phagocytosis rate, whose mechanism remains unclear.

The highly conserved CWI pathway was found to be constitutively activated in the *Δcdc50* mutant, along with the calcineurin stress response pathway. Under normal conditions, the CWI pathway is suppressed to a basal expression level to cover the regular demands of cell wall biogenesis [37]. However, once cell wall damage occurs, the cell surface sensors recognize it and deliver the signal to GEFs, which subsequently activate GTPases, PKCs, and MAPKs by phosphorylation at specific sites. Eventually, the pathway leads to the upregulated expression of effectors associated with cell wall biogenesis, such as β-(1,3)-glucan synthases Fks1 ~ 2 and chitin synthases Chs1 ~ 5, to restore cell wall integrity. The calcineurin pathway is involved in various stress responses, working via upregulation of calcium channels to activate downstream effectors [53]. Given the budding block, thicker cell wall and weakened resistance to stressors and antifungal agents associated with loss of Cdc50, we considered this abnormal hyperactivation of response pathways as a consequence of response dysregulation caused by mislocalization and malfunction of certain key regulators, rather than cell wall damage. This is similar to the situation in *C. neoformans* [30], in which the authors observed hyperactivation of the calcineurin pathway and an abnormal increase in the cellular calcium level in the *Δcdc50* mutant, which was considered to be responsible for increased sensitivity to caspofungin. In agreement, this hypersensitivity was reversed by disrupting a mechanosensitive calcium channel, Crm1, via restoring the calcium hemostasis.

Herein, we investigated the general role played by Cdc50 in cellular processes. We determined that Cdc50 has an extensive influence on yeast budding, drug resistance, cell wall integrity and macrophage uptake of *C. glabrata*. To study the underlying mechanisms, further experiments, e.g., determining the localization of significant accessory proteins, are needed. The importance of, and high conversation among fungi lipid flippases also offers potential targets for new drug research. In fact, a newly designed antifungal drug targeting the P4-ATPase function, the “AW9-Ma peptide”, has been reported to restore *C. neoformans*’ sensitivity to caspofungin [54]. Considering the lack of newly-developed clinical antifungal drugs, these studies are particularly meaningful.

## Conclusion

In the present study, we investigated the function of lipid flippase subunit Cdc50 in *C. glabrata* by constructing a *Δcdc50* null mutant. Loss of Cdc50 led to difficulty in yeast budding, hypersensitivity to azoles, caspofungin and cell wall stressors. Further experiments indicated hyperactivation of the cell wall integrity pathway in the *Δcdc50* mutant, which resulted in elevated major contents in the cell wall. The *Δcdc50* mutant also exhibited weakened virulence in nematode survival tests. This work highlights the importance of lipid flippase, and offers potential targets for new drug research.

## Materials and methods

### Strains and media

The *Candida glabrata* strains and plasmids used in this study are listed in Table 3. All strains were routinely maintained at 30 ℃ in yeast-peptone-dextrose (YPD) medium (1% yeast extract, 2% peptone, and 2% glucose) or on solid YPD medium (liquid YPD medium with 1.5% agar). YPD plates containing 100 μg/ml nourseothricin and 1000 μg/ml hygromycin were used when excising the NAT and hygR selective markers, respectively. Antifungal drugs were obtained from MCE (Monmouth Junction, NJ, USA) and Sigma-Aldrich (St. Louis, MO, USA).

**Table 3:** Strains and plasmids used in this study

Strain and plasmids	Genotype or description	Source or reference
ATCC2001	*Candida glabrata* ATCC2001 (CBS138) strain	ATCC
pYC44	The empty backbone for yeasts, *NAT*	Addgene
*Δcdc50*	ATCC2001 (*Δcdc50::NAT*)	This study
*Δcdc50* + *CDC50*	ATCC2001 (*Δcdc50::NAT*, pCN-HygR-Cdc50)	This study
pCN-PDC1-GFP	The integrating vector of *C. glabrata*, *NAT*, *GFP*	Addgene
pCN-HygR-Cdc50	pCN-PDC1-GFP (*Δnat::HygR*, *Δgfp::Cdc50*)	This study
ATCC22019	*Candida parapsilosis* ATCC22019 strain	ATCC

### Construction of the *C. glabrata Δcdc50* null mutant and complemented strains

To knockout the *Cdc50* gene in *C. glabrata*, an NAT cassette containing a nourseothricin resistance marker was used for homologous recombination, as described previously [55]. Briefly, the upstream and downstream regions of *Cdc50* were amplified using the DNA of strain ATCC2001 as the template and primers Cdc50-UP and Cdc50-DOWN, and the NAT marker was amplified using plasmid pYC44 and primers NAT-Cdc50 (the reverse complement sequences are marked in lower case in Table 4). After fusion PCR, a knockout cassette was transformed into the ATCC2001 strain, as described previously by Zhao et al. [55]. *Δcdc50* transformants that could grow on plates containing 100 μg/ml nourseothricin were selected and their genotypes were confirmed using PCR with primers y-incdc50 and y-outcdc50. Next, to generate a reintegration strain, plasmid pCN-HygR-Cdc50 with a hygromycin resistance marker and the full-length *Cdc50* gene was constructed from pCN-PDC1-GFP using an In-fusion cloning kit (Takara, Shiga, Japan). After the transformation of pCN-hygR-Cdc50, colonies able to grow on a YPD plate with 1000 μg/ml hygromycin were selected as complemented strains, with further confirmation using qRT-PCR.

**Table 4:** Primers used in this study

Primer name	Sequence (5’ → 3’)
Cdc50-UP-F	AGTGAAGAGCAGCAACTCCC
Cdc50-UP-R	cccggacagccgctaggaggtTCTGGCTCAGCCTACAGAGT
Cdc50-DOWN-F	ccgtagcccgatagtcccgagACACCTGATGCTGGTGGTAAT
Cdc50-DOWN-R	ATCGGAGGTCCAGCTCATCT
NAT-Cdc50-F	acctcctagcggctgtccgggGTTGTAAAACGACGGCCAGT
NAT-Cdc50-R	ctcgggactatcgggctacggAGGAAACAGCTATGACCATG
y-incdc50-F	CACGCCAATTAGGAGGTGGT
y-incdc50-R	GCTAGCAAGGCCAGATGGAA
y-outcdc50-F	AGTGAAGAGCAGCAACTCCC
y-outcdc50-R	ACCACCAGCATCAGGTGTTA
RT-ACT1-F	TTCCAGCCTTCTACGTTTCC
RT-ACT1-R	TCTACCAGCAAGGTCGATTC
RT-SLT2-F	GAGGCAAGCGAGGAGACTAC
RT-SLT2-R	CGCATCTGTTAGTGCTTGCC
RT-RLM1-F	GTCACCGTAAGCAGAAGGCT
RT-RLM1-R	TCTTGAAGCGTAAAGCGGGT
RT-SWI4-F	TACGTCATCTGGCATGGCTC
RT-SWI4-R	TCCAATGGCACCCAAGTACC
RT-SWI6-F	AACCCGTTTAGCCTCCACTG
RT-SWI6-R	TCACTTGCAAGCTCGTGTCT
RT-FKS1-F	TTGTCGACGGTCGTTACGTT
RT-FKS1-R	GTAGGCAGCTGGTGGTTGAT
RT-FKS2-F	CTGGTGTTGGCAATGGGTTG
RT-FKS2-R	AGCTGGGTATGGGTCGTTTG
RT-CHS3-F	AATGCCTGAGAAGGCCAGAC
RT-CHS3-R	ATCCTTTCCTGCAGCAGACC

Note: Lower case letters indicate the reverse complement sequences

### Cell growth, cell cycle distribution and budding assays

Yeast growing overnight in YPD medium at 30 ℃ were resuspended in new YPD medium and adjusted to 0.05 OD at 600 nm. After the concentration adjustments, the yeast was incubated at 30 ℃, and their OD values were recorded after 2, 4, 6, 8, 10, and 24 h to construct a cell growth curve. For cell cycle examinations, yeast grown overnight was washed and incubated in new YPD medium for 4 h. Yeast in mid-log phase was smeared, fixed, Gram stained, and observed using optical microscopy at a 100 × magnification. At least 300 yeast cells in each group were counted and classified as yeast with no bud, small-budded yeast, and large-budded yeast, to determine the cell cycle distribution as shown in Table 1. The entire experiments were repeated three times.

### Fluorescent staining of actin

Actin staining was performed using Actin-Tracker Green dye (KeyGEN, Nanjing, China), following the manufacturer's instructions. Briefly, mid-log phase yeast was collected and washed three times with phosphate-buffered saline (PBS). After being fixed in 4% paraformaldehyde solution for 10 min, the yeast was blocked in PBS buffer containing 1% bovine serum albumin (BSA) and then stained using Actin-Tracker Green solution for 20 min in the dark. Then, the yeast was washed three times with PBS and observed using fluorescent microscopy with an Axio Scope A1 microscope (Zeiss, Oberkochen, Germany), with typical field views of each group being photographed.

### Antifungal susceptibility tests and assays for stress responses

For the spot assays, yeast grown overnight in YPD medium at 30 ℃ were resuspended and grown in new YPD medium for 4 h. Mid-log phase cells were adjusted to 0.1 OD at 600 nm, serially diluted by tenfold, and 10 μl of each dilution was spotted onto YPD plates containing different antifungal agents. Phenotypes were recorded and photographed after growing at 30 ℃ for 36 h. The reference method for broth dilution antifungal susceptibility testing was followed according to the CLSI M27-S3 guidelines. Briefly, drugs were double diluted 10 times and added to 96-well plates containing mid-log phase yeast resuspended in RPMI1640 medium, with the *Candida parapsilosis* reference strain ATCC22019 used for quality control. The 50% minimum inhibitory concentrations (MIC50) of each strain were read after a 48-h incubation at 37 ℃. The experiments were repeated three times.

### Chitin, mannan and β-1,3-glucan measurements

For cell wall component assessments, FITC conjugated lectin from ConA (Sigma Aldrich), CFW (Sigma-Aldrich) and Aniline Blue (Sigma-Aldrich) were used to estimate total cell wall mannan, chitin and β-1,3-glucan contents, respectively. To further investigate the exposure of inner layer materials on the cell surface, FITC-conjugated WGA (Sigma-Aldrich) and anti-β-1,3-glucan mouse monoclonal antibodies (anti-glucan Ab) (Biosupplies, Yagoona, Australia) combined with goat anti-mouse IgG second antibodies-FITC (Abcam, Cambridge, MA, USA) were used to estimate the exposed mannan and β-(1,3)-glucan contents, respectively. Briefly, mid-log phase yeast cells were collected and washed twice with PBS, and then incubated with different stains in the dark for 30 min. After washing twice with fluorescence-activated cell sorting (FACS buffer), the yeast was analyzed using a FACSCanto II flow cytometer (BD Biosciences, San Jose, CA, USA). The β-1,3-glucan measurements were performed as described by Lee and Kim [56]: after washing with PBS buffer, the yeast cells were incubated in 1 M NaOH at 80 ℃ for 30 min. Then, 300 µl of Aniline Blue mix (40: 21: 59 of 0.1% aniline blue, 1 N HCl, and 1 M glycine/NaOH (pH 9.5)) was added to the tubes, incubated at 50 ℃ for 30 min and at room temperature for another 30 min. The fluorescent density was analyzed using a BioTek Synergy Neo2 multimode microplate reader (Berten Instruments, Montpelier, VT, USA). For β-(1,3)-glucan exposure estimation, the procedure was carried out as described by Chen et al. [40]: besides the incubation and washing, a preliminary block was needed before staining to avoid potential non-specific binding. The raw data acquired were then analyzed using FlowJo software (v.10.8.1; FlowJo LLC, Ashland, OR, USA).

### *Candida*-macrophage interaction assays

The *Candida*-macrophage interaction assays were performed as described by Hu et al. [29]. Briefly, the monocyte-like cell line THP-1 was grown in RPMI1640 medium containing 10% fetal bovine serum at 37 ℃ with 5% CO_2_. The THP-1 cells were then adjusted to a concentration of 1 × 10^6^ cells/ml and added to 24-well plates (0.5 ml per well) with fresh medium containing 100 ng/ml phorbol-12-myristate-13-acetate (Sigma-Aldrich) for differentiation and adherence induction. After 24 h of stimulation, the cells were washed and relaxed overnight before the phagocytosis assay. Mid-log phase yeast cells were collected as described above and adjusted to 0.5 OD at 600 nm. After being diluted tenfold with RPMI1640 medium, the yeast was added to 12-well plates and co-incubated with THP-1 cells for 2 h at 37 ℃ with 5% CO_2_. For the phagocytosis colony counting, after a 2-h incubation, the cells were washed three times with PBS buffer to wash away extracellular yeast. While one plate was ready for digestion, another plate was prepared for a further 4-h incubation after washing to assess the survival of the intracellular yeast. To lyse the macrophages and release the yeast inside, 0.5% Triton X-100 solution was added to the 12-well plates. After a 5-min incubation, the lysate was centrifugated at 4000 × g for 5 min, then resuspended in PBS and diluted 1000-fold. The diluent was spread on YPD plates, cultured at 30 ℃ for 48 h, and the colonies were counted to estimate the number of intracellular yeast cells. Meanwhile, after a 2-h incubation, the THP-1 cells were washed three times with PBS buffer, followed by being brushed using cytology brushes and Gram stained for microscopic yeast counting. At least 200 THP-1 cells in every group were observed to count the intracellular yeast cells. For cytokine measurements, after a 4-h co-incubation of THP-1 and yeast cells, the supernatants were collected, centrifugated at 10,000 × g for 5 min, and then the cytokines were analyzed using chemiluminescent immunoassays with the Immulite1000 system (Siemens, Munich, Germany). THP-1 cells without exposure to *C. glabrata* were used to measure the baseline level of secreted cytokines. The phagocytosis assays were repeated three times.

### Survival assays of *Caenorhabditis elegans*

To investigate the virulence of different *C. glabrata* strains, the infection model organism *Caenorhabditis elegans* was used, with *Escherichia coli* strain OP50 as a quality control. The protocol followed the instructions of Zhao et al. [55]. Briefly, after washing with M9 buffer three times, *C. elegans* (strain glp-4(bn2) I) at the L4 stage were transferred to brain heart infusion (BHI) medium plates containing yeast or to nematode growth medium (NGM) plates containing OP50 cells. After being incubated at 25 °C for 3 h, the nematodes were washed three times with BHI medium to remove attached microorganisms. About 60 nematodes were then transferred to each BHI (or NGM) plate, with each group having four identical plates to assure data reliability. The plates were incubated at 25 °C, and nematode survival was recorded every 24 h until all the nematodes were dead. Nematodes were regarded dead if they did not respond to mechanical stimuli, and dead nematodes were instantly removed from the plates throughout the experiment.

### Quantitative real-time reverse transcription PCR and transcriptome sequencing analysis

The approach used for qRT-PCR followed the method of Zhao et al. [55]. In short, *C. glabrata* cells in mid-log phase were collected, washed and frozen; then total RNA was isolated using a yeast RNAiso kit (Takara), followed by treatment with gRNA Eraser (Takara) to remove residual genomic DNA. Next, the PrimeScript RT Reagent Kit (Takara) was used to reverse transcribe the purified RNA into cDNA. The mRNA levels of the genes of interest were detected using quantitative real-time PCR (qPCR) with the 7300 Real-Time PCR System (Applied Biosystems, Beijing, China) and TB Green Premix EX Taq (Tli RNaseH Plus, ROX plus) (Takara) according to the manufacturer’s instructions. Primers used for qPCR are listed in Table 2 and *ACT1* (encoding actin) served as an endogenous control. Relative changes in gene expression were calculated using the 2^−ΔΔCT^ method [57].

For transcriptome sequencing, the protocol was as follows. First, total RNA was extracted from mid-log phase yeast using the TRIzol Reagent (Invitrogen, Waltham, MA, USA) following the manufacturer’s instructions, with removal of genomic DNA using DNase I (Takara). After confirming the quality of RNA, a RNA-seq transcriptome library was established following the instructions of the TruSeq RNA sample preparation kit (Illumina, San Diego, CA, USA). Briefly, mRNA was isolated and fragmented. Then, double-stranded cDNA was synthesized using a SuperScript ds-cDNA synthesis kit (Invitrogen). The synthesized cDNA was size selected and PCR amplified for 15 cycles. After being quantified using a TBS380 fluorometer (Turner Biosystems, Sunnyvale, CA, USA), the paired-end RNA-seq sequencing library was sequenced using an Illumina HiSeq xten/NovaSeq 6000 sequencer. After trimming and quality control using SeqPrep (https://github.com/jstjohn/SeqPrep) and Sickle (https://github.com/najoshi/sickle), clean reads were separately aligned to the reference genome with orientation mode using HISAT2 software [58]. The mapped reads of each sample were assembled by StringTie [59]. To identify DEGs between two different samples, the expression level of each transcript was calculated according to the transcripts per million reads (TPM) method. RSEM [60] was used to quantify gene abundances. Essentially, differential expression analysis was performed using DESeq2 [61], and genes with |log2 fold change (FC)|> 1 and Q value ≤ 0.05 were considered to be significantly differentially expressed. In addition, functional-enrichment analysis using Gene Ontology (GO) was performed to identify which DEGs were significantly enriched in GO terms and metabolic pathways, at a Bonferroni-corrected *P*-value ≤ 0.05 compared with the whole-transcriptome background. GO functional enrichment analysis were carried out by Goatools and KOBAS [62]. The DEG heatmap was generated by the heatmap package of the R software (https://www.R-project.org/) based on the results of GO enrichment.

## Data Availability

The RNA sequencing data discussed in this study have been deposited in NCBI's Gene Expression Omnibus and are accessible through GEO Series accession number GSE217218(https://www.ncbi.nlm.nih.gov/geo/query/acc.cgi?acc=GSE217218). For reviewer access, please use the following secure token: stwzouukxjgplgl.

## Abbreviations

BHI: Brain heart infusion medium
BSA: Bovine serum albumin
CFW: Calcofluor White
CFU: Colony-forming unit
CLSI: Clinical and Laboratory Standards Institute
ConA: Concanavalin A
CR: Congo Red
CSF: Caspofungin
CWI: Cell wall integrity
DEG: Differential expression gene
ER: Endoplasmic reticulum
FBS: Fetal bovine serum
FLC: Fluconazole
FITC: Fluorescein isothiocyanate
GO: Gene Ontology
ICZ: Itraconazole
MAPK: Mitogen-activated protein kinase
MIC50: 50% Minimum inhibitory concentration
NGM: Nematode growth medium
OD: Optical density
PBS: Phosphate-buffered saline
PC: Phosphatidylcholine
PE: Phosphatidylethanolamine
PKC: Protein kinase C
PS: Phosphoserine
qRT-PCR: Quantitative real-time reverse transcription PCR
SDS: Sodium dodecyl sulfonate
TGN: Trans-Golgi network
TPM: Transcripts per million reads
VCZ: Voriconazole
WGA: Wheat germ agglutinin
WT: Wild-type
YPD: Yeast extract peptone dextrose medium
